# *N*′-[(*E*)-4-Chloro­benzyl­idene]-2-(4-isobutyl­phen­yl)propanohydrazide

**DOI:** 10.1107/S1600536809015906

**Published:** 2009-05-07

**Authors:** Hoong-Kun Fun, Chin Sing Yeap, K. V. Sujith, B. Kalluraya

**Affiliations:** a X-ray Crystallography Unit, School of Physics, Universiti Sains Malaysia, 11800 USM, Penang, Malaysia; b Department of Studies in Chemistry, Mangalore University, Mangalagangotri, Mangalore 574 199, India

## Abstract

The asymmetric unit of title compound, C_20_H_23_ClN_2_O, consists of two crystallographically independent mol­ecules (*A* and *B*) in which the orientations of the 4-isobutyl­phenyl units are different. The isobutyl group of mol­ecule *B* is disordered over two positions with occupancies of 0.850 (5) and 0.150 (5). The dihedral angle between the two benzene rings is 88.70 (9)° in mol­ecule *A* and 89.38 (9)° in mol­ecule *B*. The independent mol­ecules are linked together into chains along [100] by N—H⋯O and C—H⋯O hydrogen bonds, and by C—H⋯π inter­actions. In the chain, N—H⋯O and C—H⋯O hydrogen bonds generate *R*_2_^1^(6) ring motifs. In addition, C—H⋯N hydrogen bonds are observed. The presence of pseudosymmetry in the structure suggests the higher symmetry space group *Pbca* but attempts to refine the structure in this space group resulted in high *R* (0.119) and *wR* (0.296) values.

## Related literature

For general background and biological applications of hydrazone compounds, see: Kawail *et al.* (2005 ▶); Klasser & Epstein, (2005 ▶); Sridhar & Perumal (2003 ▶); Bedia *et al.* (2006 ▶); Rollas *et al.* (2002 ▶); Terzioglu & Gürsoy, (2003 ▶). For bond-length data, see: Allen *et al.* (1987 ▶). For hydrogen-bond motifs, see: Bernstein *et al.* (1995 ▶). For the crystal structure of the bromo analogue, see: Fun *et al.* (2009 ▶). For the stability of the temperature controller used for the data collection, see: Cosier & Glazer (1986 ▶).
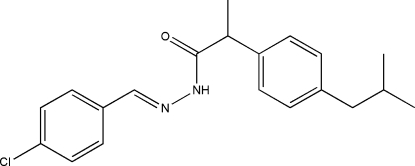


## Experimental

### 

#### Crystal data

C_20_H_23_ClN_2_O
*M* *_r_* = 342.85
Orthorhombic, 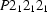

*a* = 9.1405 (1) Å
*b* = 11.9260 (2) Å
*c* = 33.3487 (5) Å
*V* = 3635.33 (9) Å^3^
*Z* = 8
Mo *K*α radiation
μ = 0.22 mm^−1^
*T* = 100 K
0.56 × 0.18 × 0.15 mm

#### Data collection

Bruker SMART APEXII CCD area-detector diffractometer
Absorption correction: multi-scan (**SADABS**; Bruker, 2005 ▶) *T*_min_ = 0.887, *T*_max_ = 0.967
26510 measured reflections
10652 independent reflections
8888 reflections with *I* > 2σ(*I*)
*R*_int_ = 0.033

#### Refinement

*R*[*F* ^2^ > 2σ(*F* ^2^)] = 0.046
*wR*(*F* ^2^) = 0.118
*S* = 1.02
10652 reflections
453 parameters
H-atom parameters constrained
Δρ_max_ = 0.28 e Å^−3^
Δρ_min_ = −0.26 e Å^−3^
Absolute structure: Flack (1983 ▶), 4712 Friedel pairs
Flack parameter: 0.10 (5)

### 

Data collection: *APEX2* (Bruker, 2005 ▶); cell refinement: *SAINT* (Bruker, 2005 ▶); data reduction: *SAINT*; program(s) used to solve structure: *SHELXTL* (Sheldrick, 2008 ▶); program(s) used to refine structure: *SHELXTL*; molecular graphics: *SHELXTL*; software used to prepare material for publication: *SHELXTL* and *PLATON* (Spek, 2009 ▶).

## Supplementary Material

Crystal structure: contains datablocks global, I. DOI: 10.1107/S1600536809015906/ci2793sup1.cif

Structure factors: contains datablocks I. DOI: 10.1107/S1600536809015906/ci2793Isup2.hkl

Additional supplementary materials:  crystallographic information; 3D view; checkCIF report

## Figures and Tables

**Table 1:** Selected torsion angles (°)

C20*A*—C9*A*—C10*A*—C11*A*	159.65 (17)
C20*A*—C9*A*—C10*A*—C15*A*	−21.5 (3)
C20*B*—C9*B*—C10*B*—C11*B*	−140.01 (18)
C20*B*—C9*B*—C10*B*—C15*B*	43.2 (2)

**Table 2:** Hydrogen-bond geometry (Å, °)

*D*—H⋯*A*	*D*—H	H⋯*A*	*D*⋯*A*	*D*—H⋯*A*
N2*A*—H1*NA*⋯O1*B*	0.86	2.02	2.821 (2)	155
C7*A*—H7*AA*⋯O1*B*	0.93	2.54	3.313 (2)	140
C12*A*—H12*A*⋯*Cg*1	0.93	2.75	3.632 (2)	159
N2*B*—H1*NB*⋯O1*A*^i^	0.86	2.02	2.832 (2)	157
C7*B*—H7*BA*⋯O1*A*^i^	0.93	2.47	3.256 (2)	143
C12*B*—H12*B*⋯*Cg*2^i^	0.93	2.65	3.462 (2)	146
C20*A*—H20*B*⋯N1*B*^ii^	0.96	2.57	3.504 (3)	164

Symmetry codes: (i) 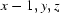; (ii) 
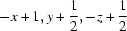
. *Cg*1 and *Cg*2 are the centroids of the C1*B*–C6*B* and C1*A*–C6*A* rings, respectively.

**Fig. 1.**
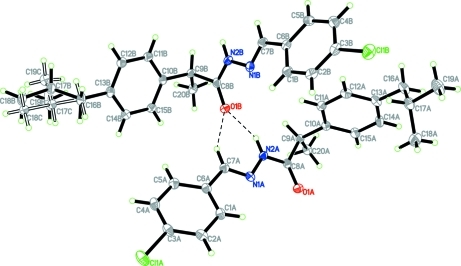
The asymmetric unit of the title compound, with atom labels and 50% probability displacement ellipsoids for non-H atoms. Hydrogen bonds are shown as dashed lines. Both disorder components are shown.

**Fig. 2.**
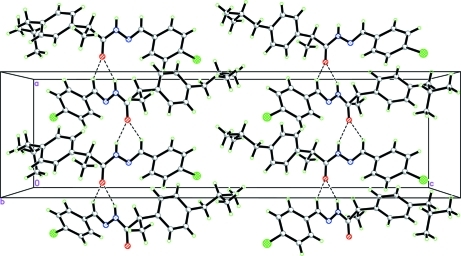
The crystal packing of of the title compound, viewed down the b axis, showing chains along the [100]. Hydrogen bonds are shown as dashed lines. Only the major disorder component is shown.

## supplementary crystallographic information

### Comment

Non-steroidal anti-inflammatory drugs (NSAIDs) such as ibuprofen are widely used in the treatment of pain and inflammation (Kawail *et al.*, 2005; Klasser & Epstein, 2005). Aryl hydrazones are important building blocks for the synthesis of a variety of heterocyclic compounds such as pyrazolines and pyrazoles (Sridhar & Perumal, 2003). Aryl hydrazones have been most conveniently synthesized by the reaction of aryl hydrazines with carbonyl compounds. Hydrazones possessing an azometine -NHN=CH- proton constitute an important class of compounds for new drug development. Hydrazones have been demonstrated to possess antimicrobial, anticonvulsant, analgesic, antiinflammatory, antiplatelet, antitubercular, anticancer and antitumoral activities (Bedia *et al.*, 2006; Rollas *et al.*, 2002; Terzioglu & Gürsoy, 2003). These observations have been the guide for the development of new hydrazones that possess varied biological activities. Prompted by these observations, the title compoud was synthesized and its crystal structure is reported here.

The asymmetric unit of title compound (Fig. 1), consists of two crystallographically independent molecules, *A* and *B*, in which the orientations of the 4-isobutylphenyl units are different (Table 1). Bond lengths (Allen *et al.*, 1987) and angles are within normal ranges and are comparable with those in the bromo analogue (Fun *et al.*, 2009). The dihedral angle between the two benzene rings is 88.70 (9)° in molecule *A* and 89.38 (9)° in molecule *B*. The molecule *A* is linked to the molecule *B* by intermolecular C7A—H7AA···O1B and N2A—H1NA···O1B hydrogen bonds, generating an *R*_2_^1^(6) ring motif (Bernstein *et al.*, 1995) and by an C—H···π interaction.

In the crystal structure, the independent molecules are linked together into chains along the [100] (Fig. 2) by N—H···O and C—H···O hydrogen bonds, and C—H···π interactions (Table 2). In addition, C—H···N hydrogen bonds are observed.

### Experimental

A mixture of 2-[4-(2-methylpropyl)phenyl]propanehydrazide (0.01 mol) and 4-chlorobenzaldehyde (0.01 mol) in ethanol (30 ml) along with 3 drops of concentrated sulphuric acid was refluxed for 1 h. Excess ethanol was removed from the reaction mixture under reduced pressure. The solid product obtained was filtered, washed with ethanol and dried. Single crystals suitable for X-ray analysis were obtained by slow evaporation of an ethanol solution (yield 87%; m.p.430-433 K).

### Refinement

The isopropyl group of molecule *B* is disordered over two positions with occupancies of 0.850 (5) and 0.150 (5). H atoms were positioned geometrically and refined using a riding model, with N-H = 0.86 Å, C-H = 0.93–0.98 Å and *U*_iso_(H) = 1.2-1.5 *U*_eq_(N, C). A rotating-group model was applied for the methyl groups. The presence of pseudo-symmetry in the structure suggests a higher symmetry space group *Pbca*. But attempts to refine the structure in the space group *Pbca* resulted in a more disordered model with high R (0.119) and wR (0.296) values.

### Crystal data

**Table:**

C_20_H_23_ClN_2_O	*F*(000) = 1456
*M**_r_* = 342.85	*D*_x_ = 1.253 Mg m^−^^3^
Orthorhombic, *P*2_1_2_1_2_1_	Mo *K*α radiation, λ = 0.71073 Å
Hall symbol: P 2ac 2ab	Cell parameters from 7618 reflections
*a* = 9.1405 (1) Å	θ = 2.4–30.0°
*b* = 11.9260 (2) Å	µ = 0.22 mm^−^^1^
*c* = 33.3487 (5) Å	*T* = 100 K
*V* = 3635.33 (9) Å^3^	Block, colourless
*Z* = 8	0.56 × 0.18 × 0.15 mm

### Data collection

**Table:**

Bruker SMART APEXII CCD area-detector diffractometer	10652 independent reflections
Radiation source: fine-focus sealed tube	8888 reflections with *I* > 2σ(*I*)
graphite	*R*_int_ = 0.033
φ and ω scans	θ_max_ = 30.1°, θ_min_ = 1.2°
Absorption correction: multi-scan (*SADABS*; Bruker, 2005)	*h* = −12→12
*T*_min_ = 0.887, *T*_max_ = 0.967	*k* = −13→16
26510 measured reflections	*l* = −46→46

### Refinement

**Table:**

Refinement on *F*^2^	Secondary atom site location: difference Fourier map
Least-squares matrix: full	Hydrogen site location: inferred from neighbouring sites
*R*[*F*^2^ > 2σ(*F*^2^)] = 0.046	H-atom parameters constrained
*wR*(*F*^2^) = 0.118	*w* = 1/[σ^2^(*F*_o_^2^) + (0.0575*P*)^2^ + 0.6608*P*] where *P* = (*F*_o_^2^ + 2*F*_c_^2^)/3
*S* = 1.02	(Δ/σ)_max_ = 0.001
10652 reflections	Δρ_max_ = 0.28 e Å^−^^3^
453 parameters	Δρ_min_ = −0.25 e Å^−^^3^
0 restraints	Absolute structure: Flack (1983), 4712 Friedel pairs
Primary atom site location: structure-invariant direct methods	Flack parameter: 0.10 (5)

### Special details

**Table:**

Experimental. The crystal was placed in the cold stream of an Oxford Cyrosystems Cobra open-flow nitrogen cryostat (Cosier & Glazer, 1986) operating at 100.0 (1)K.
Geometry. All esds (except the esd in the dihedral angle between two l.s. planes) are estimated using the full covariance matrix. The cell esds are taken into account individually in the estimation of esds in distances, angles and torsion angles; correlations between esds in cell parameters are only used when they are defined by crystal symmetry. An approximate (isotropic) treatment of cell esds is used for estimating esds involving l.s. planes.
Refinement. Refinement of F^2^ against ALL reflections. The weighted R-factor wR and goodness of fit S are based on F^2^, conventional R-factors R are based on F, with F set to zero for negative F^2^. The threshold expression of F^2^ > 2sigma(F^2^) is used only for calculating R-factors(gt) etc. and is not relevant to the choice of reflections for refinement. R-factors based on F^2^ are statistically about twice as large as those based on F, and R- factors based on ALL data will be even larger.

### Fractional atomic coordinates and isotropic or equivalent isotropic displacement parameters (Å^2^)

**Table:**

	*x*	*y*	*z*	*U*_iso_*/*U*_eq_	Occ. (<1)
Cl1A	0.85958 (7)	−0.01289 (4)	0.079925 (15)	0.03026 (12)	
O1A	0.87919 (14)	0.49658 (12)	0.27831 (4)	0.0223 (3)	
N1A	0.72753 (18)	0.36296 (13)	0.22621 (5)	0.0175 (3)	
N2A	0.66284 (18)	0.44256 (13)	0.25054 (4)	0.0172 (3)	
H1NA	0.5699	0.4534	0.2493	0.021*	
C1A	0.8193 (2)	0.16989 (16)	0.17979 (5)	0.0191 (4)	
H1AA	0.8688	0.1789	0.2039	0.023*	
C2A	0.8699 (2)	0.09272 (16)	0.15182 (6)	0.0215 (4)	
H2AA	0.9526	0.0497	0.1571	0.026*	
C3A	0.7946 (2)	0.08102 (16)	0.11585 (6)	0.0215 (4)	
C4A	0.6700 (2)	0.14204 (16)	0.10760 (6)	0.0227 (4)	
H4AA	0.6206	0.1324	0.0835	0.027*	
C5A	0.6195 (2)	0.21807 (16)	0.13586 (6)	0.0207 (4)	
H5AA	0.5346	0.2587	0.1307	0.025*	
C6A	0.6943 (2)	0.23433 (16)	0.17187 (5)	0.0181 (4)	
C7A	0.6421 (2)	0.32052 (15)	0.19980 (5)	0.0184 (4)	
H7AA	0.5456	0.3447	0.1983	0.022*	
C8A	0.7450 (2)	0.50325 (15)	0.27615 (5)	0.0164 (4)	
C9A	0.6584 (2)	0.58104 (15)	0.30371 (5)	0.0161 (3)	
H9AA	0.5618	0.5933	0.2917	0.019*	
C10A	0.6374 (2)	0.51916 (15)	0.34336 (5)	0.0173 (3)	
C11A	0.5317 (2)	0.43554 (16)	0.34657 (6)	0.0186 (4)	
H11A	0.4730	0.4188	0.3246	0.022*	
C12A	0.5130 (2)	0.37699 (16)	0.38200 (6)	0.0207 (4)	
H12A	0.4411	0.3219	0.3834	0.025*	
C13A	0.5986 (2)	0.39827 (16)	0.41551 (5)	0.0204 (4)	
C14A	0.7039 (2)	0.48227 (17)	0.41241 (6)	0.0233 (4)	
H14A	0.7624	0.4987	0.4345	0.028*	
C15A	0.7232 (2)	0.54207 (16)	0.37695 (5)	0.0212 (4)	
H15A	0.7941	0.5979	0.3756	0.025*	
C16A	0.5822 (2)	0.32870 (16)	0.45308 (6)	0.0247 (4)	
H16A	0.4792	0.3226	0.4597	0.030*	
H16B	0.6305	0.3668	0.4751	0.030*	
C17A	0.6470 (2)	0.21025 (16)	0.44874 (6)	0.0235 (4)	
H17A	0.5996	0.1738	0.4258	0.028*	
C18A	0.8110 (2)	0.21425 (19)	0.44058 (7)	0.0314 (5)	
H18A	0.8470	0.1395	0.4364	0.047*	
H18B	0.8602	0.2472	0.4631	0.047*	
H18C	0.8292	0.2586	0.4171	0.047*	
C19A	0.6155 (3)	0.14012 (19)	0.48615 (6)	0.0357 (5)	
H19A	0.6571	0.0668	0.4830	0.053*	
H19B	0.5116	0.1337	0.4898	0.053*	
H19C	0.6579	0.1759	0.5092	0.053*	
C20A	0.7355 (2)	0.69465 (16)	0.30705 (6)	0.0203 (4)	
H20A	0.6858	0.7403	0.3265	0.030*	
H20B	0.7338	0.7315	0.2815	0.030*	
H20C	0.8350	0.6834	0.3153	0.030*	
Cl1B	0.35621 (6)	−0.01924 (4)	0.418134 (16)	0.03065 (12)	
O1B	0.38166 (14)	0.50246 (12)	0.22302 (4)	0.0224 (3)	
N1B	0.23212 (17)	0.37147 (13)	0.27639 (5)	0.0183 (3)	
N2B	0.16586 (18)	0.44848 (14)	0.25107 (4)	0.0189 (3)	
H1NB	0.0729	0.4590	0.2523	0.023*	
C1B	0.3149 (2)	0.17172 (17)	0.32054 (6)	0.0210 (4)	
H1BA	0.3605	0.1792	0.2958	0.025*	
C2B	0.3619 (2)	0.09038 (16)	0.34692 (6)	0.0214 (4)	
H2BA	0.4372	0.0419	0.3398	0.026*	
C3B	0.2958 (2)	0.08155 (16)	0.38423 (6)	0.0217 (4)	
C4B	0.1802 (2)	0.15040 (17)	0.39501 (6)	0.0235 (4)	
H4BA	0.1361	0.1433	0.4200	0.028*	
C5B	0.1313 (2)	0.22994 (16)	0.36785 (6)	0.0207 (4)	
H5BA	0.0522	0.2753	0.3745	0.025*	
C6B	0.1994 (2)	0.24292 (15)	0.33062 (5)	0.0178 (4)	
C7B	0.1467 (2)	0.33044 (16)	0.30332 (5)	0.0192 (4)	
H7BA	0.0511	0.3564	0.3055	0.023*	
C8B	0.2472 (2)	0.50689 (16)	0.22453 (5)	0.0177 (4)	
C9B	0.1567 (2)	0.57335 (16)	0.19427 (5)	0.0184 (4)	
H9BA	0.0596	0.5875	0.2057	0.022*	
C10B	0.1401 (2)	0.49807 (15)	0.15762 (5)	0.0166 (3)	
C11B	0.0116 (2)	0.43596 (16)	0.15239 (6)	0.0204 (4)	
H11B	−0.0652	0.4442	0.1704	0.025*	
C12B	−0.0020 (2)	0.36179 (16)	0.12028 (6)	0.0201 (4)	
H12B	−0.0889	0.3223	0.1170	0.024*	
C13B	0.1114 (2)	0.34555 (15)	0.09307 (5)	0.0189 (4)	
C14B	0.2380 (2)	0.40909 (17)	0.09833 (6)	0.0221 (4)	
H14B	0.3150	0.4009	0.0803	0.027*	
C15B	0.2516 (2)	0.48440 (17)	0.12987 (5)	0.0221 (4)	
H15B	0.3369	0.5263	0.1324	0.026*	
C16B	0.0976 (2)	0.26297 (16)	0.05913 (5)	0.0225 (4)	
H16C	0.0608	0.1938	0.0698	0.027*	0.850 (5)
H16D	0.1935	0.2485	0.0486	0.027*	0.850 (5)
H16E	0.0156	0.2152	0.0647	0.027*	0.150 (5)
H16F	0.1835	0.2167	0.0593	0.027*	0.150 (5)
C17B	−0.0010 (3)	0.2988 (2)	0.02462 (7)	0.0242 (6)	0.850 (5)
H17B	−0.1012	0.3044	0.0348	0.029*	0.850 (5)
C18B	0.0013 (3)	0.2096 (3)	−0.00823 (9)	0.0302 (7)	0.850 (5)
H18D	−0.0631	0.2315	−0.0296	0.045*	0.850 (5)
H18E	−0.0307	0.1392	0.0027	0.045*	0.850 (5)
H18F	0.0990	0.2018	−0.0184	0.045*	0.850 (5)
C19B	0.0411 (5)	0.4117 (2)	0.00756 (8)	0.0498 (10)	0.850 (5)
H19D	0.0312	0.4681	0.0279	0.075*	0.850 (5)
H19E	−0.0219	0.4295	−0.0146	0.075*	0.850 (5)
H19F	0.1407	0.4093	−0.0015	0.075*	0.850 (5)
C17C	0.0720 (16)	0.3086 (10)	0.0176 (4)	0.014 (2)*	0.150 (5)
H17C	0.1601	0.3497	0.0095	0.017*	0.150 (5)
C18C	0.0557 (15)	0.2076 (14)	−0.0106 (5)	0.014 (2)*	0.15
H18G	0.1379	0.1583	−0.0073	0.021*	0.150 (5)
H18H	0.0518	0.2332	−0.0379	0.021*	0.150 (5)
H18I	−0.0328	0.1680	−0.0043	0.021*	0.150 (5)
C19C	−0.0562 (16)	0.3885 (12)	0.0151 (4)	0.023 (3)*	0.150 (5)
H19G	−0.0360	0.4539	0.0309	0.035*	0.150 (5)
H19H	−0.1425	0.3522	0.0251	0.035*	0.150 (5)
H19I	−0.0714	0.4101	−0.0123	0.035*	0.150 (5)
C20B	0.2306 (2)	0.68628 (17)	0.18538 (6)	0.0234 (4)	
H20D	0.2272	0.7327	0.2089	0.035*	
H20E	0.1802	0.7230	0.1638	0.035*	
H20F	0.3307	0.6738	0.1779	0.035*	

### Atomic displacement parameters (Å^2^)

**Table:**

	*U*^11^	*U*^22^	*U*^33^	*U*^12^	*U*^13^	*U*^23^
Cl1A	0.0455 (3)	0.0200 (2)	0.0253 (2)	−0.0009 (2)	0.0077 (2)	−0.0070 (2)
O1A	0.0167 (6)	0.0280 (7)	0.0221 (7)	0.0015 (6)	−0.0005 (5)	−0.0034 (6)
N1A	0.0202 (7)	0.0167 (7)	0.0157 (7)	0.0004 (6)	0.0024 (6)	−0.0009 (6)
N2A	0.0159 (8)	0.0183 (8)	0.0173 (7)	0.0035 (6)	−0.0023 (6)	−0.0022 (6)
C1A	0.0247 (10)	0.0162 (9)	0.0163 (9)	−0.0019 (8)	−0.0005 (8)	0.0012 (7)
C2A	0.0259 (10)	0.0167 (9)	0.0219 (9)	0.0005 (8)	0.0050 (8)	0.0019 (7)
C3A	0.0316 (10)	0.0146 (9)	0.0183 (9)	−0.0041 (8)	0.0048 (8)	−0.0033 (8)
C4A	0.0300 (10)	0.0201 (10)	0.0179 (9)	−0.0050 (8)	−0.0008 (8)	−0.0021 (8)
C5A	0.0222 (10)	0.0190 (9)	0.0211 (9)	−0.0042 (8)	−0.0002 (8)	0.0009 (7)
C6A	0.0209 (10)	0.0168 (9)	0.0167 (8)	−0.0033 (7)	0.0015 (7)	0.0017 (7)
C7A	0.0190 (8)	0.0182 (9)	0.0181 (8)	−0.0018 (8)	0.0001 (7)	0.0011 (7)
C8A	0.0188 (9)	0.0162 (9)	0.0143 (8)	0.0017 (7)	−0.0011 (7)	0.0016 (7)
C9A	0.0171 (8)	0.0167 (8)	0.0146 (8)	0.0022 (7)	−0.0001 (7)	0.0007 (7)
C10A	0.0193 (8)	0.0153 (8)	0.0171 (8)	0.0028 (7)	−0.0001 (7)	−0.0008 (7)
C11A	0.0188 (9)	0.0174 (9)	0.0197 (9)	0.0013 (7)	−0.0020 (7)	−0.0021 (7)
C12A	0.0201 (9)	0.0160 (9)	0.0259 (10)	−0.0024 (7)	0.0032 (8)	−0.0016 (8)
C13A	0.0272 (9)	0.0166 (9)	0.0175 (8)	0.0009 (7)	0.0051 (7)	0.0006 (7)
C14A	0.0286 (10)	0.0252 (10)	0.0161 (8)	−0.0054 (8)	−0.0029 (7)	−0.0001 (7)
C15A	0.0235 (9)	0.0208 (9)	0.0193 (9)	−0.0073 (8)	−0.0002 (7)	−0.0004 (7)
C16A	0.0336 (10)	0.0217 (9)	0.0189 (9)	0.0003 (8)	0.0056 (8)	0.0010 (8)
C17A	0.0304 (10)	0.0198 (9)	0.0203 (9)	0.0000 (8)	0.0009 (8)	0.0018 (7)
C18A	0.0295 (11)	0.0303 (11)	0.0344 (11)	0.0014 (9)	−0.0031 (9)	0.0033 (9)
C19A	0.0532 (14)	0.0260 (11)	0.0278 (11)	0.0021 (10)	0.0060 (10)	0.0099 (9)
C20A	0.0242 (9)	0.0162 (9)	0.0205 (9)	0.0001 (7)	0.0007 (7)	0.0021 (7)
Cl1B	0.0338 (3)	0.0233 (2)	0.0348 (3)	0.0022 (2)	−0.0057 (2)	0.0096 (2)
O1B	0.0165 (6)	0.0305 (8)	0.0202 (6)	0.0027 (6)	−0.0005 (5)	0.0010 (6)
N1B	0.0198 (8)	0.0185 (8)	0.0164 (7)	0.0032 (6)	−0.0025 (6)	−0.0019 (6)
N2B	0.0157 (8)	0.0225 (9)	0.0186 (8)	0.0040 (7)	−0.0030 (6)	0.0008 (6)
C1B	0.0202 (9)	0.0222 (10)	0.0207 (9)	0.0012 (8)	−0.0011 (8)	−0.0035 (8)
C2B	0.0200 (9)	0.0164 (9)	0.0279 (10)	0.0009 (8)	−0.0007 (8)	−0.0032 (8)
C3B	0.0226 (9)	0.0162 (9)	0.0262 (10)	−0.0016 (8)	−0.0058 (8)	0.0020 (8)
C4B	0.0255 (10)	0.0238 (10)	0.0213 (9)	−0.0026 (8)	0.0000 (8)	0.0005 (8)
C5B	0.0188 (9)	0.0187 (9)	0.0245 (9)	0.0019 (8)	0.0019 (8)	−0.0006 (7)
C6B	0.0173 (9)	0.0167 (9)	0.0194 (9)	0.0003 (7)	−0.0032 (7)	0.0000 (7)
C7B	0.0176 (8)	0.0195 (9)	0.0206 (9)	0.0036 (8)	−0.0024 (8)	−0.0019 (7)
C8B	0.0193 (9)	0.0188 (9)	0.0150 (8)	0.0004 (8)	−0.0013 (7)	−0.0045 (7)
C9B	0.0169 (8)	0.0196 (9)	0.0187 (8)	0.0016 (8)	−0.0021 (7)	−0.0001 (7)
C10B	0.0189 (8)	0.0173 (9)	0.0138 (7)	0.0013 (7)	−0.0022 (7)	0.0021 (7)
C11B	0.0182 (9)	0.0234 (10)	0.0196 (9)	−0.0026 (8)	−0.0001 (7)	0.0011 (8)
C12B	0.0208 (9)	0.0185 (9)	0.0210 (9)	−0.0063 (7)	−0.0015 (7)	0.0018 (7)
C13B	0.0232 (9)	0.0166 (8)	0.0171 (8)	0.0013 (7)	−0.0040 (7)	0.0012 (7)
C14B	0.0202 (8)	0.0282 (10)	0.0181 (9)	0.0001 (8)	0.0010 (7)	−0.0026 (8)
C15B	0.0177 (8)	0.0285 (10)	0.0201 (9)	−0.0043 (8)	−0.0012 (7)	−0.0011 (8)
C16B	0.0272 (9)	0.0192 (9)	0.0211 (9)	0.0010 (8)	−0.0054 (7)	−0.0023 (7)
C17B	0.0253 (14)	0.0268 (13)	0.0203 (11)	0.0026 (10)	−0.0055 (10)	−0.0049 (9)
C18B	0.0330 (15)	0.0326 (15)	0.0251 (13)	−0.0039 (14)	−0.0069 (13)	−0.0105 (11)
C19B	0.101 (3)	0.0250 (14)	0.0231 (13)	0.0006 (17)	−0.0188 (17)	−0.0003 (11)
C20B	0.0252 (10)	0.0195 (9)	0.0255 (10)	−0.0006 (8)	−0.0045 (8)	−0.0016 (8)

### Geometric parameters (Å, °)

**Table:**

Cl1A—C3A	1.744 (2)	C1B—C2B	1.378 (3)
O1A—C8A	1.231 (2)	C1B—C6B	1.395 (3)
N1A—C7A	1.281 (2)	C1B—H1BA	0.93
N1A—N2A	1.382 (2)	C2B—C3B	1.387 (3)
N2A—C8A	1.348 (2)	C2B—H2BA	0.93
N2A—H1NA	0.86	C3B—C4B	1.386 (3)
C1A—C2A	1.390 (3)	C4B—C5B	1.386 (3)
C1A—C6A	1.402 (3)	C4B—H4BA	0.93
C1A—H1AA	0.93	C5B—C6B	1.397 (3)
C2A—C3A	1.390 (3)	C5B—H5BA	0.93
C2A—H2AA	0.93	C6B—C7B	1.467 (3)
C3A—C4A	1.379 (3)	C7B—H7BA	0.93
C4A—C5A	1.387 (3)	C8B—C9B	1.527 (3)
C4A—H4AA	0.93	C9B—C10B	1.524 (2)
C5A—C6A	1.396 (3)	C9B—C20B	1.536 (3)
C5A—H5AA	0.93	C9B—H9BA	0.98
C6A—C7A	1.467 (3)	C10B—C15B	1.386 (3)
C7A—H7AA	0.93	C10B—C11B	1.400 (3)
C8A—C9A	1.527 (2)	C11B—C12B	1.394 (3)
C9A—C10A	1.526 (2)	C11B—H11B	0.93
C9A—C20A	1.531 (3)	C12B—C13B	1.392 (3)
C9A—H9AA	0.98	C12B—H12B	0.93
C10A—C11A	1.393 (3)	C13B—C14B	1.394 (3)
C10A—C15A	1.394 (2)	C13B—C16B	1.506 (2)
C11A—C12A	1.383 (3)	C14B—C15B	1.389 (3)
C11A—H11A	0.93	C14B—H14B	0.93
C12A—C13A	1.387 (3)	C15B—H15B	0.93
C12A—H12A	0.93	C16B—C17C	1.507 (12)
C13A—C14A	1.393 (3)	C16B—C17B	1.523 (3)
C13A—C16A	1.510 (2)	C16B—H16C	0.96
C14A—C15A	1.392 (3)	C16B—H16D	0.96
C14A—H14A	0.93	C16B—H16E	0.96
C15A—H15A	0.93	C16B—H16F	0.96
C16A—C17A	1.539 (3)	C17B—C19B	1.511 (4)
C16A—H16A	0.97	C17B—C18B	1.528 (3)
C16A—H16B	0.97	C17B—H17B	0.98
C17A—C18A	1.524 (3)	C18B—H18D	0.96
C17A—C19A	1.529 (3)	C18B—H18E	0.96
C17A—H17A	0.98	C18B—H18F	0.96
C18A—H18A	0.96	C19B—H19D	0.96
C18A—H18B	0.96	C19B—H19E	0.96
C18A—H18C	0.96	C19B—H19F	0.96
C19A—H19A	0.96	C17C—C19C	1.513 (19)
C19A—H19B	0.96	C17C—C18C	1.535 (19)
C19A—H19C	0.96	C17C—H17C	0.98
C20A—H20A	0.96	C18C—H18G	0.96
C20A—H20B	0.96	C18C—H18H	0.96
C20A—H20C	0.96	C18C—H18I	0.96
Cl1B—C3B	1.740 (2)	C19C—H19G	0.96
O1B—C8B	1.231 (2)	C19C—H19H	0.96
N1B—C7B	1.287 (2)	C19C—H19I	0.96
N1B—N2B	1.387 (2)	C20B—H20D	0.96
N2B—C8B	1.350 (2)	C20B—H20E	0.96
N2B—H1NB	0.86	C20B—H20F	0.96
			
C7A—N1A—N2A	114.48 (16)	C1B—C2B—H2BA	120.3
C8A—N2A—N1A	120.17 (15)	C3B—C2B—H2BA	120.3
C8A—N2A—H1NA	119.9	C4B—C3B—C2B	121.32 (18)
N1A—N2A—H1NA	119.9	C4B—C3B—Cl1B	118.85 (16)
C2A—C1A—C6A	120.52 (18)	C2B—C3B—Cl1B	119.82 (15)
C2A—C1A—H1AA	119.7	C5B—C4B—C3B	118.79 (18)
C6A—C1A—H1AA	119.7	C5B—C4B—H4BA	120.6
C1A—C2A—C3A	118.74 (19)	C3B—C4B—H4BA	120.6
C1A—C2A—H2AA	120.6	C4B—C5B—C6B	120.88 (18)
C3A—C2A—H2AA	120.6	C4B—C5B—H5BA	119.6
C4A—C3A—C2A	121.88 (18)	C6B—C5B—H5BA	119.6
C4A—C3A—Cl1A	118.90 (15)	C1B—C6B—C5B	118.92 (17)
C2A—C3A—Cl1A	119.22 (16)	C1B—C6B—C7B	122.13 (17)
C3A—C4A—C5A	118.98 (18)	C5B—C6B—C7B	118.95 (17)
C3A—C4A—H4AA	120.5	N1B—C7B—C6B	120.31 (18)
C5A—C4A—H4AA	120.5	N1B—C7B—H7BA	119.8
C4A—C5A—C6A	120.82 (19)	C6B—C7B—H7BA	119.8
C4A—C5A—H5AA	119.6	O1B—C8B—N2B	123.68 (18)
C6A—C5A—H5AA	119.6	O1B—C8B—C9B	122.43 (17)
C5A—C6A—C1A	119.03 (18)	N2B—C8B—C9B	113.76 (16)
C5A—C6A—C7A	118.98 (18)	C10B—C9B—C8B	106.14 (15)
C1A—C6A—C7A	121.98 (17)	C10B—C9B—C20B	113.93 (15)
N1A—C7A—C6A	121.01 (18)	C8B—C9B—C20B	110.15 (15)
N1A—C7A—H7AA	119.5	C10B—C9B—H9BA	108.8
C6A—C7A—H7AA	119.5	C8B—C9B—H9BA	108.8
O1A—C8A—N2A	123.87 (17)	C20B—C9B—H9BA	108.8
O1A—C8A—C9A	121.34 (17)	C15B—C10B—C11B	118.13 (17)
N2A—C8A—C9A	114.77 (15)	C15B—C10B—C9B	122.11 (17)
C10A—C9A—C8A	107.03 (14)	C11B—C10B—C9B	119.69 (17)
C10A—C9A—C20A	115.01 (15)	C12B—C11B—C10B	120.44 (18)
C8A—C9A—C20A	110.07 (15)	C12B—C11B—H11B	119.8
C10A—C9A—H9AA	108.2	C10B—C11B—H11B	119.8
C8A—C9A—H9AA	108.2	C13B—C12B—C11B	121.48 (18)
C20A—C9A—H9AA	108.2	C13B—C12B—H12B	119.3
C11A—C10A—C15A	117.96 (17)	C11B—C12B—H12B	119.3
C11A—C10A—C9A	119.99 (16)	C12B—C13B—C14B	117.44 (17)
C15A—C10A—C9A	122.04 (17)	C12B—C13B—C16B	121.24 (17)
C12A—C11A—C10A	120.86 (18)	C14B—C13B—C16B	121.32 (17)
C12A—C11A—H11A	119.6	C15B—C14B—C13B	121.42 (17)
C10A—C11A—H11A	119.6	C15B—C14B—H14B	119.3
C11A—C12A—C13A	121.73 (18)	C13B—C14B—H14B	119.3
C11A—C12A—H12A	119.1	C10B—C15B—C14B	121.04 (17)
C13A—C12A—H12A	119.1	C10B—C15B—H15B	119.5
C12A—C13A—C14A	117.48 (17)	C14B—C15B—H15B	119.5
C12A—C13A—C16A	120.79 (17)	C13B—C16B—C17C	117.9 (5)
C14A—C13A—C16A	121.67 (17)	C13B—C16B—C17B	115.73 (16)
C15A—C14A—C13A	121.26 (17)	C13B—C16B—H16C	108.2
C15A—C14A—H14A	119.4	C17C—C16B—H16C	126.7
C13A—C14A—H14A	119.4	C17B—C16B—H16C	108.4
C14A—C15A—C10A	120.71 (17)	C13B—C16B—H16D	108.4
C14A—C15A—H15A	119.6	C17B—C16B—H16D	108.3
C10A—C15A—H15A	119.6	H16C—C16B—H16D	107.5
C13A—C16A—C17A	112.86 (15)	C13B—C16B—H16E	107.9
C13A—C16A—H16A	109.0	C17C—C16B—H16E	105.8
C17A—C16A—H16A	109.0	H16D—C16B—H16E	132.7
C13A—C16A—H16B	109.0	C13B—C16B—H16F	107.7
C17A—C16A—H16B	109.0	C17C—C16B—H16F	109.8
H16A—C16A—H16B	107.8	C17B—C16B—H16F	130.5
C18A—C17A—C19A	110.38 (18)	H16E—C16B—H16F	107.3
C18A—C17A—C16A	111.51 (17)	C19B—C17B—C16B	112.6 (2)
C19A—C17A—C16A	110.67 (16)	C19B—C17B—C18B	110.3 (2)
C18A—C17A—H17A	108.1	C16B—C17B—C18B	109.8 (2)
C19A—C17A—H17A	108.1	C19B—C17B—H17B	108.0
C16A—C17A—H17A	108.1	C16B—C17B—H17B	108.0
C17A—C18A—H18A	109.5	C18B—C17B—H17B	108.0
C17A—C18A—H18B	109.5	C16B—C17C—C19C	113.4 (10)
H18A—C18A—H18B	109.5	C16B—C17C—C18C	107.2 (10)
C17A—C18A—H18C	109.5	C19C—C17C—C18C	112.7 (11)
H18A—C18A—H18C	109.5	C16B—C17C—H17C	107.8
H18B—C18A—H18C	109.5	C19C—C17C—H17C	107.8
C17A—C19A—H19A	109.5	C18C—C17C—H17C	107.8
C17A—C19A—H19B	109.5	C17C—C18C—H18G	109.5
H19A—C19A—H19B	109.5	C17C—C18C—H18H	109.5
C17A—C19A—H19C	109.5	H18G—C18C—H18H	109.5
H19A—C19A—H19C	109.5	C17C—C18C—H18I	109.5
H19B—C19A—H19C	109.5	H18G—C18C—H18I	109.5
C9A—C20A—H20A	109.5	H18H—C18C—H18I	109.5
C9A—C20A—H20B	109.5	C17C—C19C—H19G	109.5
H20A—C20A—H20B	109.5	C17C—C19C—H19H	109.5
C9A—C20A—H20C	109.5	H19G—C19C—H19H	109.5
H20A—C20A—H20C	109.5	C17C—C19C—H19I	109.5
H20B—C20A—H20C	109.5	H19G—C19C—H19I	109.5
C7B—N1B—N2B	114.31 (16)	H19H—C19C—H19I	109.5
C8B—N2B—N1B	120.03 (16)	C9B—C20B—H20D	109.5
C8B—N2B—H1NB	120.0	C9B—C20B—H20E	109.5
N1B—N2B—H1NB	120.0	H20D—C20B—H20E	109.5
C2B—C1B—C6B	120.68 (18)	C9B—C20B—H20F	109.5
C2B—C1B—H1BA	119.7	H20D—C20B—H20F	109.5
C6B—C1B—H1BA	119.7	H20E—C20B—H20F	109.5
C1B—C2B—C3B	119.36 (18)		
			
C7A—N1A—N2A—C8A	−171.84 (16)	C2B—C3B—C4B—C5B	−0.6 (3)
C6A—C1A—C2A—C3A	0.2 (3)	Cl1B—C3B—C4B—C5B	−179.42 (16)
C1A—C2A—C3A—C4A	−1.3 (3)	C3B—C4B—C5B—C6B	−1.7 (3)
C1A—C2A—C3A—Cl1A	178.34 (15)	C2B—C1B—C6B—C5B	−0.5 (3)
C2A—C3A—C4A—C5A	0.7 (3)	C2B—C1B—C6B—C7B	179.92 (18)
Cl1A—C3A—C4A—C5A	−178.97 (15)	C4B—C5B—C6B—C1B	2.2 (3)
C3A—C4A—C5A—C6A	1.0 (3)	C4B—C5B—C6B—C7B	−178.22 (18)
C4A—C5A—C6A—C1A	−2.1 (3)	N2B—N1B—C7B—C6B	176.26 (16)
C4A—C5A—C6A—C7A	176.25 (17)	C1B—C6B—C7B—N1B	−24.7 (3)
C2A—C1A—C6A—C5A	1.4 (3)	C5B—C6B—C7B—N1B	155.66 (18)
C2A—C1A—C6A—C7A	−176.85 (18)	N1B—N2B—C8B—O1B	−5.5 (3)
N2A—N1A—C7A—C6A	−179.59 (16)	N1B—N2B—C8B—C9B	170.38 (15)
C5A—C6A—C7A—N1A	−158.07 (18)	O1B—C8B—C9B—C10B	82.4 (2)
C1A—C6A—C7A—N1A	20.2 (3)	N2B—C8B—C9B—C10B	−93.60 (18)
N1A—N2A—C8A—O1A	3.6 (3)	O1B—C8B—C9B—C20B	−41.4 (2)
N1A—N2A—C8A—C9A	−175.07 (15)	N2B—C8B—C9B—C20B	142.63 (17)
O1A—C8A—C9A—C10A	−82.3 (2)	C8B—C9B—C10B—C15B	−78.2 (2)
N2A—C8A—C9A—C10A	96.38 (18)	C20B—C9B—C10B—C11B	−140.01 (18)
O1A—C8A—C9A—C20A	43.3 (2)	C20B—C9B—C10B—C15B	43.2 (2)
N2A—C8A—C9A—C20A	−137.99 (16)	C8B—C9B—C10B—C11B	98.61 (19)
C8A—C9A—C10A—C11A	−77.7 (2)	C15B—C10B—C11B—C12B	0.7 (3)
C20A—C9A—C10A—C11A	159.65 (17)	C9B—C10B—C11B—C12B	−176.29 (17)
C8A—C9A—C10A—C15A	101.1 (2)	C10B—C11B—C12B—C13B	1.2 (3)
C20A—C9A—C10A—C15A	−21.5 (3)	C11B—C12B—C13B—C14B	−2.1 (3)
C15A—C10A—C11A—C12A	−0.2 (3)	C11B—C12B—C13B—C16B	178.27 (17)
C9A—C10A—C11A—C12A	178.72 (17)	C12B—C13B—C14B—C15B	1.1 (3)
C10A—C11A—C12A—C13A	−0.5 (3)	C16B—C13B—C14B—C15B	−179.27 (18)
C11A—C12A—C13A—C14A	0.8 (3)	C11B—C10B—C15B—C14B	−1.7 (3)
C11A—C12A—C13A—C16A	−176.36 (18)	C9B—C10B—C15B—C14B	175.23 (17)
C12A—C13A—C14A—C15A	−0.5 (3)	C13B—C14B—C15B—C10B	0.8 (3)
C16A—C13A—C14A—C15A	176.63 (18)	C12B—C13B—C16B—C17C	104.7 (7)
C13A—C14A—C15A—C10A	−0.1 (3)	C14B—C13B—C16B—C17C	−75.0 (7)
C11A—C10A—C15A—C14A	0.5 (3)	C12B—C13B—C16B—C17B	74.0 (3)
C9A—C10A—C15A—C14A	−178.40 (18)	C14B—C13B—C16B—C17B	−105.7 (2)
C12A—C13A—C16A—C17A	73.5 (2)	C13B—C16B—C17B—C19B	54.1 (3)
C14A—C13A—C16A—C17A	−103.5 (2)	C17C—C16B—C17B—C19B	−47.5 (10)
C13A—C16A—C17A—C18A	61.8 (2)	C13B—C16B—C17B—C18B	177.4 (2)
C13A—C16A—C17A—C19A	−174.92 (18)	C17C—C16B—C17B—C18B	75.9 (10)
C7B—N1B—N2B—C8B	172.53 (17)	C13B—C16B—C17C—C19C	−52.6 (12)
C6B—C1B—C2B—C3B	−1.7 (3)	C17B—C16B—C17C—C19C	39.9 (10)
C1B—C2B—C3B—C4B	2.2 (3)	C13B—C16B—C17C—C18C	−177.6 (7)
C1B—C2B—C3B—Cl1B	−178.93 (15)	C17B—C16B—C17C—C18C	−85.1 (13)

### Hydrogen-bond geometry (Å, °)

**Table:**

*D*—H···*A*	*D*—H	H···*A*	*D*···*A*	*D*—H···*A*
N2A—H1NA···O1B	0.86	2.02	2.821 (2)	155
C7A—H7AA···O1B	0.93	2.54	3.313 (2)	140
C12A—H12A···Cg1	0.93	2.75	3.632 (2)	159
N2B—H1NB···O1A^i^	0.86	2.02	2.832 (2)	157
C7B—H7BA···O1A^i^	0.93	2.47	3.256 (2)	143
C12B—H12B···Cg2^i^	0.93	2.65	3.462 (2)	146
C20A—H20B···N1B^ii^	0.96	2.57	3.504 (3)	164

Symmetry codes: (i) *x*−1, *y*, *z*; (ii) −*x*+1, *y*+1/2, −*z*+1/2.
